# Human amniotic fluid stem cell injection therapy for urethral sphincter regeneration in an animal model

**DOI:** 10.1186/1741-7015-10-94

**Published:** 2012-08-21

**Authors:** Bum Soo Kim, So Young Chun, Jong Kil Lee, Hyun Ju Lim, Jae-sung Bae, Ho-Yun Chung, Anthony Atala, Shay Soker, James J Yoo, Tae Gyun Kwon

**Affiliations:** 1 Department of Urology, School of Medicine, Kyungpook National University, Daegu, Korea; 2 Joint Institute for Regenerative Medicine, Kyungpook National University Hospital, Daegu, Korea; 3 Department of Physiology, School of Medicine, Kyungpook National University, Daegu, Korea; 4 Department of Plastic and Reconstructive Surgery, School of Medicine, Kyungpook National University, Daegu, Korea; 5 Wake Forest Institute for Regenerative Medicine, Wake Forest University School of Medicine, Winston-Salem, NC, USA

**Keywords:** urinary incontinence, amniotic fluid, stem cells

## Abstract

**Background:**

Stem cell injection therapies have been proposed to overcome the limited efficacy and adverse reactions of bulking agents. However, most have significant limitations, including painful procurement, requirement for anesthesia, donor site infection and a frequently low cell yield. Recently, human amniotic fluid stem cells (hAFSCs) have been proposed as an ideal cell therapy source. In this study, we investigated whether periurethral injection of hAFSCs can restore urethral sphincter competency in a mouse model.

**Methods:**

Amniotic fluids were collected and harvested cells were analyzed for stem cell characteristics and *in vitro *myogenic differentiation potency. Mice underwent bilateral pudendal nerve transection to generate a stress urinary incontinence (SUI) model and received either periurethral injection of hAFSCs, periurethral injection of Plasma-Lyte (control group), or underwent a sham (normal control group).

For *in vivo *cell tracking, cells were labeled with silica-coated magnetic nanoparticles containing rhodamine B isothiocyanate (MNPs@SiO2 (RITC)) and were injected into the urethral sphincter region (n = 9). Signals were detected by optical imaging. Leak point pressure and closing pressure were recorded serially after injection.

Tumorigenicity of hAFSCs was evaluated by implanting hAFSCs into the subcapsular space of the kidney, followed two weeks later by retrieval and histologic analysis.

**Results:**

Flow activated cell sorting showed that hAFSCs expressed mesenchymal stem cell (MSC) markers, but no hematopoietic stem cell markers. Induction of myogenic differentiation in the hAFSCs resulted in expression of *PAX7 *and *MYOD *at Day 3, and *DYSTROPHIN *at Day 7. The nanoparticle-labeled hAFSCs could be tracked *in vivo *with optical imaging for up to 10 days after injection. Four weeks after injection, the mean LPP and CP were significantly increased in the hAFSC-injected group compared with the control group. Nerve regeneration and neuromuscular junction formation of injected hAFSCs *in vivo *was confirmed with expression of neuronal markers and acetylcholine receptor. Injection of hAFSCs caused no *in vivo *host CD8 lymphocyte aggregation or tumor formation.

**Conclusions:**

hAFSCs displayed MSC characteristics and could differentiate into cells of myogenic lineage. Periurethral injection of hAFSCs into an SUI animal model restored the urethral sphincter to apparently normal histology and function, in absence of immunogenicity and tumorigenicity.

## Background

Stress urinary incontinence (SUI), defined as the involuntary leakage of urine upon physical activity, sneezing or coughing, is an embarrassing problem in women [1]. Treatment modalities for SUI include pharmacotherapy, surgery and injection of bulking agents. Surgical approaches, such as tension free vaginal tape, transobturator slings or pubovaginal slings, remain the gold standards for SUI treatment. The efficacy of pharmacotherapy for SUI has been disappointing. Attempts to avoid invasive surgery and morbidity have included the use of various injectable bulking agents, including polytetrafluoroethylene, bovine collagen, silicone particles, carbon beads and autologous fat or chondrocytes [2]. However, these procedures have had limited success and frequent adverse reactions, such as allergic and immune reactions, infection, particle migration and reabsorption of injected bulking agents [2].

Stem cell therapy has been proposed as an attractive alternative to overcome the limitations and side effects of pharmacotherapeutic procedures [3-6]. One of the most commonly used cell types are bone marrow stromal cells [4]. However, bone marrow procurement requires general or spinal anesthesia, and yields a low number of stem cells upon processing. Muscle derived stem cells and adipose derived stem cells have been proposed as alternative cell sources [5,6]. Although these cells can be obtained in large quantities under local anesthesia, procurement remains an invasive procedure with the risk of morbidity. Recently, we reported the use of human amniotic fluid stem cells (hAFSCs), which can be obtained non-invasively, have a high proliferation rate, induce immune tolerance, display embryonic stem cell properties and are able to differentiate into cells representing all three embryonic germ layers [7]. These characteristics suggest hAFSCs might be an ideal cell source for stem cell therapy applications.

The primary objective of this study was to investigate whether periurethral injection of hAFSCs results in restoration of the urethral sphincter to normal histology and function. Secondary objectives were to characterize hAFSCs stem cell properties and myogenicity *in vitro*, to develop a non-invasive method for tracking injected cells, and to evaluate *in vivo *viability, immunogenicity and tumorigenicity of transplanted hAFSCs.

## Methods

### Isolation and culture of hAFSCs

This study was approved by the Ethics Committee of Kyungpook National University School of Medicine. All participants provided informed consent. Amniotic fluids (10 mL each) were obtained from four women undergoing routine amniocentesis at a gestational age of 15 to 19 weeks. Amniotic fluids were centrifuged and supernatants discarded. Cell pellets were resuspended with Chang Medium (α-MEM, 15% embryonic stem cell-fetal bovine serum (Gibco-Invitrogen, Grand Island, NY, USA) with 18% Chang B and 2% Chang C (Irvine Scientific, Irvine, CA, USA)} in a petri dish. Non-adherent cells were discarded after one week. Adherent cells were passaged for expansion on reaching 80% confluence, and culture medium was replaced every three days.

### Characterization of hAFSCs

hAFSCs (Passage 3) were evaluated by flow cytometry with phycoerythrin (PE)- or fluorescein isothiocyanate-conjugated mouse monoclonal antibodies specific for embryonic stem cell marker SSEA4, MSC markers CD44, CD73, CD90 and CD105, hematopoietic stem cell marker CD45, and immunologic markers HLA-ABC and HLA-DR (BD Biosciences, San Jose, CA, USA), according to the manufacturer's instructions. Approximately 10,000 cells were measured using a fluorescence activated cell sorter (FACS; BD Biosciences) system equipped with the CellQuest program. A homogenous stem cell population was obtained by sorting the cells twice using C-KIT antibody (Santa Cruz Biotechnology, Santa Cruz, CA, USA) with a magnetic activated cell sorting system (MACS, Miltenyi Biotec, Bergischu Gladbach, Germany). In case #1, a cell population with high expression for C-KIT and SSEA4 as well as low expression for HLA-DR was obtained and used for subsequent studies.

### Myogenic differentiation of hAFSCs *in vitro*

The optimum myogenic condition for hAFSC induction was determined by testing three different induction media: (i) myogenic medium (DMEM, 0.5% chick embryo extract, 10% horse serum, Gibco-Invitrogen) containing 3 μM of 5-aza-20-deoxycytidine (5-azaC; Sigma-Aldrich, St. Louis, MO, USA), (ii) myogenic medium containing 5 ng/mL of transforming growth factor-β (TGF-β; Peprotech, Rocky Hill, NJ, USA), and (iii) conditioned medium (CM; collected from cultured human skeletal muscle cells). After 24-hour treatment with 5-azaC or TGF-β, cells were cultured up to a further 14 days. Cell viabilities at 1, 3, 5 and 14 days of differentiation were measured using a CCK-8 assay kit (Dojindo, Kumamoto, Japan,) according to the manufacturer's instructions. The genotypic and morphologic conversion of hAFSC into myocytes was analyzed by real-time polymerase chain reaction (PCR) and immunocytochemical (ICC) staining using routine methods. The primer sequences and antibody information are listed in Tables 1 and 2, respectively. The C2C12 cell line and human fibroblasts served as positive and negative controls for ICC staining respectively. The same experiment was repeated three times independently.

**Table 1:** Primer sequences for real-time PCR

Gene	Sequences
*PAX7*	5'-GCAAATTGCTGTCCTGCTCA 5'-TGAAAACTGGTCACATCTGCCT

*MYF5*	5'-ACCGATTCACAGCCTCGAACT 5'-TGTGTATTAGGCCCTCCTGGAA

*MYOD*	5'-ACAGCGCGGTTTTTTCCAC 5'-AACCTAGCCCCTCAAGGTTCAG

*MYOGENIN*	5'-TGGCAGGAACAAGCCTTTTC 5'-ACAGGCAGGTAGTTTTCCCCA

*MEF2*	5'-ATTCCACCAGGCAGCAAGAA 5'-GGAGTTGCTACGGAAACCACTG

*MLP*	5'-AAGGCTCTTGACAGCACGACAG 5'-TGTCCATACCCGATCCCTTTG

*β-ACTIN*	5'-ATCGTCCACCGCAAATGCT 5'-AAGCCATGCCAATCTCATCTTG

*Pax7*	5'-ACCAAGCTTTCAAGTCCGCA 5'-GCCTTACATTCTGGAGGATGGA

*Myf*	5'-CTCTGAAGGATGGACATGACGG 5'-ACTGGTCCCCAAACTCATCCTC

*MyoD*	5'-TTCCGGAGTGGCAGAAAGTTAA 5'-TCAAGTCTATGTCCCGGAGTGG

*Myogenin*	5'-TATCCGGTTCCAAAGCCTCTG 5'-GCGGCAGCTTTACAAACAACA

*Mef2*	5'-AACCCCAATCTTCTGCCACTG 5'-ATCAGACCGCCTGTGTTACCTG

*Mlp*	5'-GCTGAACAAGTTACTGAGCGGC 5'-ATTTTGCACCTCCACCCCA

*Gapdh*	5'-TGT GTCCGTCGTGGATCTGA 5'-CCTGCTTCACCACCTTCTTGA

**Table 2:** Antibody information for ICC, IHC and FACS analysis

Antibody		Company	Dilution
MYOD	Myogenic determination	Santa Cruz Biotechnology	1:300

DES	desmin	Abcam	1:300

α-SM ACTIN	alpha smooth muscle actin	Santa Cruz Biotechnology	1:300

MHC	Myosin heavy chain	Santa Cruz Biotechnology	1:300

α-ACTININ	alpha actinin	Sigma-Aldrich	1:300

CD8	cluster of differentiation 8	Chemicon	1:200

SSEA4	stage-specific embryonic antigen4	BD Pharmingen	1:20

C-KIT(CD117)	cluster of differentiation117	BD Pharmingen	1:20

CD44	cluster of differentiation44	BD Pharmingen	1:20

CD45	cluster of differentiation45	BD Pharmingen	1:20

CD73	cluster of differentiation73	BD Pharmingen	1:20

CD90	cluster of differentiation90	BD Pharmingen	1:20

CD105	cluster of differentiation105	Abcam	1:20

HLA-ABC	human leukocyte antigen-ABC	BD Pharmingen	1:20

HLA-DR	human leukocyte antigen-DR	BD Pharmingen	1:20

HuNu	human nuclear-specific	Cell Signaling	1:100

α-Bungarotoxin	α-Bungarotoxin	Invitrogen	1:100

### Generation of an incompetent urethral sphincter model

All experimental protocols were approved by the Animal Ethics Committee, Kyungpook National University School of Medicine. Female imprinting control region (ICR) mice, weighing 20 to 25 g, were obtained from Hyochang Science (Daegu, Korea). Animals were prepared for aseptic surgery under general anesthesia (isoflurane). A lower midline abdominal incision was made and the bladder and urethra were exposed. The SUI model was created using a bilateral pudendal nerve transection technique instead of crush injury of the pudendal nerve. This allowed us to confirm the functional recovery of incontinence by regeneration of the urethral sphincter and to eliminate the effect of pudendal nerve regeneration. The pudendal nerve on each side was identified and transected with microsurgical scissors under microscopic magnification (n = 30). Laparotomy was closed in layers with absorbable 4 to 0 vicryl sutures. An additional 15 mice underwent a sham operation (lower midline incision and closure) and served as normal controls.

### Injection of hAFSCs

One week after generation of the incompetent urethral sphincter model, animals were anesthetized with isoflurane and the bladder and urethra were exposed by a lower abdominal incision. The urethra was slightly retracted and hAFSCs were injected at the three and nine o'clock area of the external sphincter using a 26G Hamilton microsyringe (Hamilton Company, Reno, NV, USA). The depth of injection was determined by an experienced operator. Each injection consisted of 0.5 × 10^6 ^undifferentiated AFSCs (Passage 5) in 5 μL of Plasma-Lyte. Three experimental groups were established: a sham-operation group (Ctrl), a pudendal neurectomy without cell injection group (Cell (-)) and a pudendal neurectomy with cell injection group (Cell (+)) (n = 15 for each group). The fate of injected cells *in vivo *was traced by immunohistochemical (IHC) staining with human nuclear-specific antibody at 3, 5, 7 and 14 days after injection.

### MNPs@SiO2 labeling of hAFSCs and *in vivo *tracking

Silica-coated magnetic nanoparticles incorporating rhodamine B isothiocyanate (MNPs@SiO2 (RITC)) were kindly provided by Dr. Jae-sung Bae (Kyungpook National University, Daegu, Korea). MNPs@SiO2 (RITC) is a novel cell-tracking agent with multimodal fluorescence and magnetic properties. MNPs@SiO2 (RITC) nanoparticles become integrated into the cells by endocytosis. The nanoparticles are not shed by cells and the signal cannot be detected after cell death. When cells are proliferating or differentiating, MNPs@SiO2 (RITC) transfers into daughter cells, where each cell particle number becomes gradually reduced. MNPs@SiO2 (RITC) is biocompatible and has been used for various stem cell tracking studies [8-10]. In the present study, the optimal concentration and exposure time of MNPs@SiO_2 _(RITC) for efficient uptake of nanoparticles into AFSCs was established by incubating cells at 37°C with various concentrations (0.01, 0.05, 0.1 or 0.2 mg/mL) of MNPs@SiO_2 _(RITC) for different exposure times (24 to 72 hours). Briefly, the AFSCs (2 × 10^4 ^per chamber well) were cultured in Lab-Tek™ Chamber Slides (four-chamber mounted Permanox slides; Nunc, Rochester, NY, USA). On reaching about 60 to 70% confluence, cells were incubated with MNPs@SiO_2 _(RITC) in a 37°C/5% CO_2 _incubator. Labeling was stopped by washing the cells three times with PBS. Cells were then fixed by incubation with 4% paraformaldehyde for 20 minutes at 4°C and washed with PBS. Individual coverslips were mounted onto VECTASHIELD mounting medium with DAPI (Vector Laboratories, Burlingame, CA, USA). Cells were examined with a fluorescence microscope (Olympus BX51, Tokyo, Japan) and confocal microscope (Olympus FluoViewTM FV1000) to determine the intracellular localization of nanoparticles.

For *in vivo *cell tracking, unsorted MNPs@SiO2 (NIR-797) labeled cells (0.5 × 10^6^) were injected into the urethral sphincter region of mice (n = 9), and optical images were obtained at 3, 7, 10 and 14 days using Optix exPlore (ART, Montreal, QC, Canada) with the filter set for NIR797. Images were corrected for background and autofluorescence using non-labeled cells as controls. Acquired images were analyzed with eXplore Optix OptiView software. For optical imaging, the animal was anesthetized by intravenous injection of a Rompun (Bayer, Leverkusen, Germany):Zoletil (Virbac Australia, Milperra NSW, Australia):saline mixture (1:5.7:10; 100 μL/mouse) according to the manufacturers' guidelines.

### Measurement of leak point pressure (LPP) and closing pressure (CP)

Mice were placed under general anesthesia with ether to avoid muscle relaxation. LPP and CP were measured one, two and four weeks after cell injection using the vertical tilt/intravesical pressure clamp model of SUI as previously described [11], and the Power Lab^® ^system (AD Instruments Pty Ltd, Bella Vista NSW, Australia). Before measuring, the spinal cord was transected at the T9 to T10 level to eliminate reflex bladder activity in response to increasing intravesical pressure. This suprasacral spinal cord transection does not interfere with the spinal continence reflexes of the bladder neck and urethra [12]. Under general anesthesia, the bladder was exposed by a midline incision. A transvesical catheter with a fire-flared tip (PE-25) was inserted into the dome of the bladder and the abdominal wall and overlying skin were closed with sutures. The mice were then mounted on a tilt table and placed in the vertical position. The intravesical pressure was clamped by connecting a large 50 mL syringe reservoir to the bladder catheter and the pressure transducer using PE-50 tubing and three-way stopcocks. The intravesical pressure was increased in 1- to 3-cm H_2_O steps from 0 cm H_2_O upward until visual identification of the leak point height. The pressure at this leak point was referred to as the LPP. The intravesical pressure was then decreased in 1- to 3-cm H_2_O steps downward until the leak ceased. The pressure at this leak cessation point was taken as the CP. The averages of three consecutive LPP and CP measurements were taken as data points for each animal.

### Histological, IHC, and real-time PCR analysis

Animals were sacrificed after LPP and CP measurement and the bladder-urethra complex was removed *en bloc*. Tissue specimens were fixed in 10% buffered formalin, processed and cut into 4- to 6-μM-thick sections for routine staining with hematoxylin and eosin (H&E), and for IHC staining. The presence and migration of injected cells *in situ *were analyzed by IHC using human nuclear specific antibody (HuNu; Cell Signaling Technology, Danvers, MA, USA). Myogenic conversion of injected hAFSCs *in vivo *was confirmed with real-time PCR using human primers for myogenic lineage markers. To investigate whether injected hAFSCs induced a host myogenic response, real-time PCR analysis was performed using mouse primers for myogenic lineage markers. The neuromuscular junction formation was analyzed at Week 4 by IHC staining for the acetylcholine receptor with α-Bungarotoxin. The possibility that hAFSC injection induced host neuroregeneration was investigated by real-time PCR with mouse neurogenic-related primers.

### Immunogenicity and tumorigenicity of hAFSCs

The immunogenicity of hAFSCs was evaluated by flow cytometry to determine expression of HLA-DR on the cell surface. The *in vivo *immunosuppression effect of hAFSCs was examined by retrieving tissue one week after injection and performing IHC staining with cytotoxic T cell marker (CD8, BD Pharmigen, San Jose, CA, USA). As a negative control, human fibroblasts were injected (n = 3). Tumorigenicity was analyzed by injecting 1 × 10^6 ^hAFSCs into the subcapsular space of the kidney (n = 9). Animals were sacrificed eight weeks later and kidneys were harvested for histologic confirmation.

### Statistical analysis

Our main outcome measure was to compare differences between LPP and CP between the hAFSCs injection and control groups in the SUI animal model. We collected data from five animals in each group at one, two and four weeks after injection. The results were also compared with those of the normal control (n = 5, at each time point). Data were presented as means ± SD. Statistical analysis was undertaken with Student's *t*-test or one-way analysis of variance (ANOVA). A *P*-value of less than 0.05 was considered statistically significant. When the value was found to be significant after assessment using the ANOVA statistical test, the Tukey *post-hoc *comparison was used.

## Results

### Characterization of human amniotic fluid cells and isolation of c-kit C-KIT (+) cells

**Figure 1:**
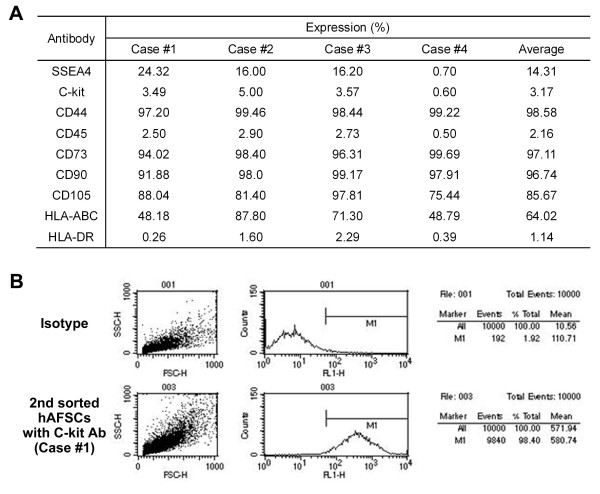
**Characterization of hAFSCs *in vitro***. (**A**) FACS analyses for expression of mesenchymal, hematopoietic and immunologic markers. hAFSCs showed strong positive expression of mesenchymal markers (CD44, CD73, CD90 and CD105), but were negative for the hematopoietic lineage marker (CD45) and MHC Class II antigen (HLA-DR). **(B) **Representative FACS image (case #1) of C-KIT (+) cell population after a double sorting procedure. Approximately 98.4% of cells were C-KIT (+).

We obtained adherent cells from all four amniotic fluid samples. FACS analysis on culture-expanded cells (Passage 3) showed the cells were strongly positive for the mesenchymal markers, CD44, CD73, CD90 and CD105; weakly positive for the embryonic marker, SSEA-4; and negative for the hematopoietic lineage marker, CD45 and MHC Class II antigen, HLA-DR (Figure 1A). Double MACS analysis showed 98.40% of the cell population in case #1 was C-KIT (+) (Figure 1B). The C-KIT (+) populations in other cases were less than 90% (data not shown). Therefore, we used the C-KIT (+) cells from case #1 for subsequent studies.

### Myogenic differentiation potential of hAFSCs *in vitro*

**Figure 2:**
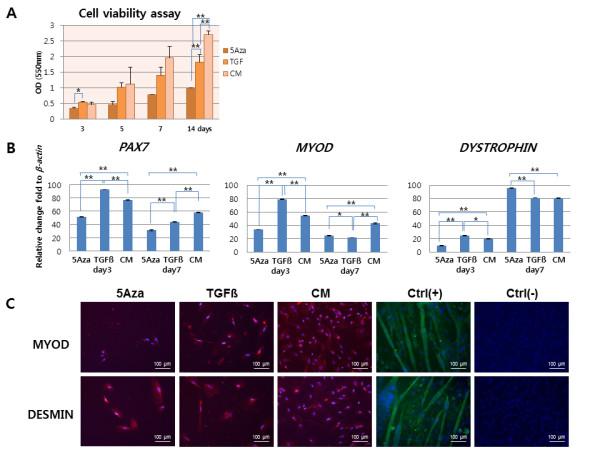
**Myogenic differentiation of hAFSCs *in vitro***. **(A) **Viability assay for cells cultured in three different myogenic induction media for 14 days. Cell viability in CM was 2.7- (*P *< 0.0001) and 1.58- (*P *< 0.0001) fold higher than that of cells cultured in medium containing 5-azaC or TGF-β respectively. (**B) **Real-time PCR analysis of expression of myogenic lineage markers in three different media at Days 3 and 7. The expression of early myogenic differentiation markers (*PAX7 *and *MYOD*) was dominant at Day 3 and the expression of the mid to late myogenic marker (*DYSTROPHIN*) became dominant at Day 7 (**P < 0.01; *P < 0.05). **(C) **Double staining using primary myogenic antibodies and DAPI through ICC analysis (200×) at Day 7. Cells were strongly positive for MYOD and DESMIN. Ctrl(+), positive control C2C12 cells; Ctrl(-), negative control with human fibroblasts.

When hAFSCs were cultured in myogenic induction media, the cells become elongated and with spindle shapes at Day 7. These findings were similar in all three groups. CCK-8 assays, performed at Day 14 showed a 2.7- (*P *< 0.0001) and 1.58- (*P *< 0.0001) fold higher cell viability for cells cultured in CM compared with cells cultured in medium containing 5-azaC or TGF-β, respectively (Figure 2A). Real-time PCR showed that expression of early myogenic differentiation markers (*PAX7 *and *MYOD*) was dominant at Day 3, whereas the mid to late myogenic marker (*DYSTROPHIN*) became dominant at Day 7. Each gene expression level varied depending on the medium type (Figure 2B). These results were confirmed by ICC staining (Figure 2C).

### *In vivo *identification of injected cells

**Figure 3:**
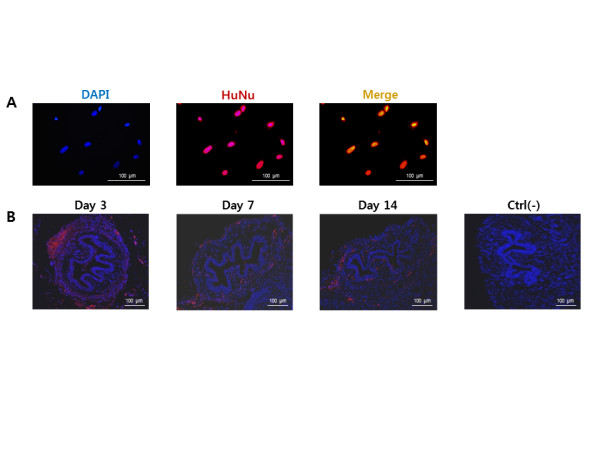
**Identification of hAFSCs *in situ *with IHC staining**. **(A) **HuNu expression of hAFSCs *in vitro *(200×). **(B) **Localization of the injected hAFSCs. At Day 3, the injected cells were detected focally at the injection site. They migrated into surrounding tissue from Day 5 and cell signals were gradually decreased from Day 7 (200×). Ctrl (-), negative control without cell treatment.

*In vitro *ICC staining with HuNu confirmed the presence of hAFSCs (Figure 3A). IHC staining confirmed focal detection of injected cells at the injection site at Day 3. Cells then migrated into the surrounding urethral sphincter region (Figure 3B). The cell signal decreased from Day 7 and had disappeared by Day 14.

### Optical imaging for *in vivo *tracking of injected cells

**Figure 4:**
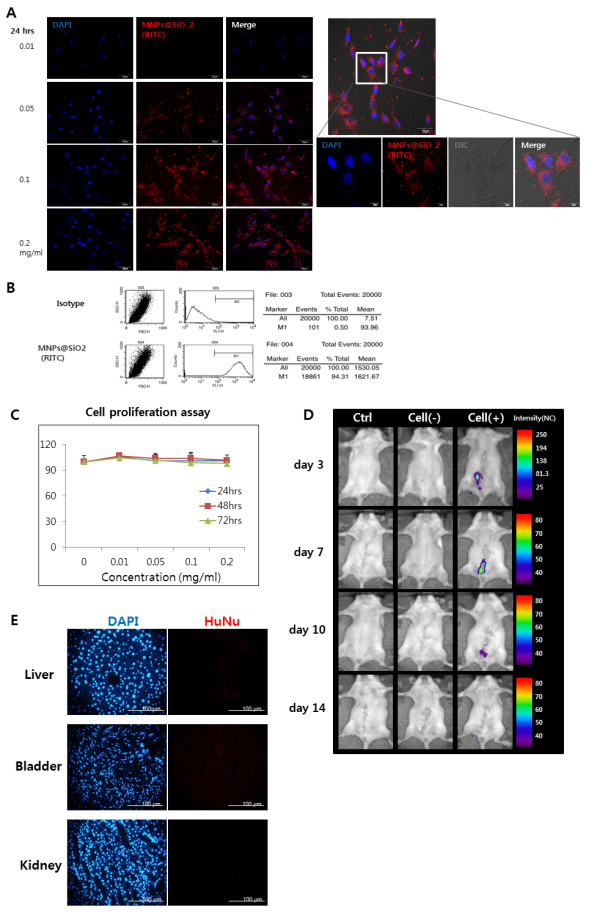
**Non-invasive cell tracking of MNPs@SiO2 (RITC) labeled hAFSCs**. **(A) **Fluorescence image of hAFSCs labeled with MNPs@SiO2 (RITC). Following treatment, nanoparticles entered the cytoplasm. The optimum concentration of MNPs@SiO2 (RITC) was 0.1 mg/mL with a treatment time of 24 hours. **(B) **FACS analysis showed labeling in 94.31% of cells. **(C) **Cell proliferation assays at various concentrations and exposure times. **(D) ***In vivo *cell tracking of nanoparticle-labeled hAFSCs. The cells could be tracked with optical imaging up to 14 days after injection. **(E) **HuNu was used to monitor hAFSCs migration into other organs. IHC staining revealed no positive expression in the liver, bladder, or kidney.

The optimum concentration and treatment time for MNPs@SiO2 (RITC) labeling of hAFSCs were 0.1 mg/mL and 24 hours, respectively (Figure 4A). The maximum labeling efficacy was 94.31% (Figure 4B). MNPs@SiO2 (RITC) uptake was uniform for each cell preparation and density. The cell viability assay confirmed that the nanoparticles did not induce cytotoxicity at a wide range of concentrations (0.05 to 0.2 mg/mL) and exposure times (up to 72 hours) (Figure 4C). Injection of MNPs@SiO2 (RITC) labeled hAFSCs into the periurethral region confirmed that optical imaging could identify these cell clusters at the injection site. The signal intensity decreased gradually until Day 10 after injection and disappeared thereafter (Figure 4D).

### LPP and CP measurement

**Figure 5:**
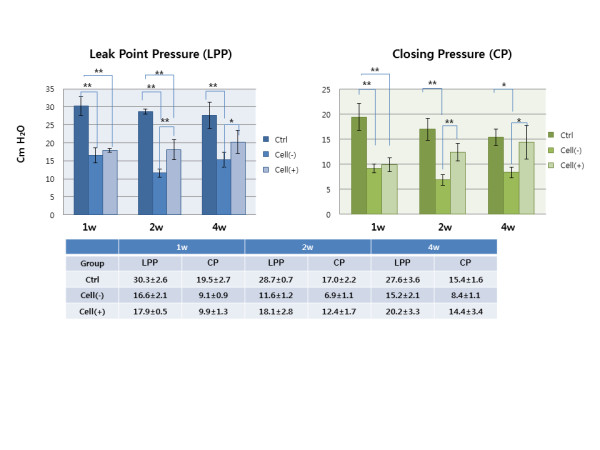
**LPP and CP measurement**. LPP and CP were measured one, two and four weeks after injection. Mean LPP and CP were significantly higher in hAFSC-injected animals than in animals without cell injection. Ctrl, positive control with sham operation; Cell (-), pudendal neurectomy without cell injection; Cell (+), pudendal neurectomy with hAFSCs injection (**P < 0.01; *P < 0.05). For LPP, Ctrl vs. Cell(-) and Cell (+) at Week 1, *P *= 0.0009 and 0.0015; Ctrl vs. Cell (-) and Cell (+) at Week 2, *P *= 0.0015 and 0.002; Cell(-) vs. Cell(+) at Week 2, *P *= 0.009; Ctrl vs. Cell(-) at Week 4, *P *= 0.0052; Cell (-) vs. Cell (+) at Week 4, *P *= 0.024. For CP, Ctrl vs. Cell (-) and Cell (+) at Week 1, *P *= 0.0016 and 0.0021; Ctrl vs. Cell (-) at Week 2, *P *= 0.0025; Cell (-) vs. Cell (+) at Week 2, *P *= 0.009; Ctrl vs. Cell (-) at Week 4, *P *= 0.0358; Cell (-) vs. Cell (+) at Week 4, *P *= 0.036.

LPP and CP were measured one, two and four weeks after injection (Figure 5). The mean LPP and CP for the Cell (+) group were similar to those of the Cell (-) group at Week 1 (17.9 ± 0.5 *vs*. 16.6 ± 2.1 and 9.9 ± 1.3 *vs*. 9.1 ± 0.9 cmH_2_O). However, the mean LPP was significantly higher for the Cell (+) group than for the Cell (-) group at Week 2 (18.1 ± 2.8 *vs*. 11.6 ± 1.2 cmH_2_O, *P *= 0.0014) and Week 4 (20.2 ± 3.3 *vs*. 15.2 ± 2.1 cmH_2_O, *P *= 0.0202). The mean CP for the Cell (+) and Cell (-) groups showed similar differences to those found for LPP at the same time points (Week 2: 12.4 ± 1.7 *vs*. 6.9 ± 1.1 cmH_2_O, *P *= 0.0001 and Week 4: 14.4 ± 3.4 *vs*. 8.4 ± 1.1 cmH_2_O, *P *= 0.0051).

### Histological, IHC and real-time PCR analysis

**Figure 6:**
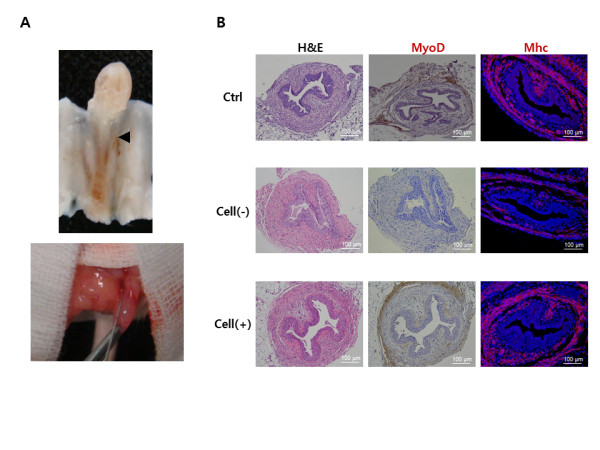
**Anatomical, histological and IHC analysis of regenerated sphincters**. **(A) **Anatomic location of the urethral sphincter and cell injection. Location of the urethral sphincter on mouse model (upper) and cell injection on the urethral sphincter area using a Hamilton microsyringe (lower). **(B) **The sphincter of hAFSCs-injected animals showed apparently normal muscle regeneration with strong MyoD and myosin heavy chain (MHC) expression. The Cell (-) group showed atrophic sphincters. Ctrl, positive control with sham operation; Cell (-), pudendal neurectomy without cell injection; Cell (+), pudendal neurectomy with hAFSCs injection (200×).

**Figure 7:**
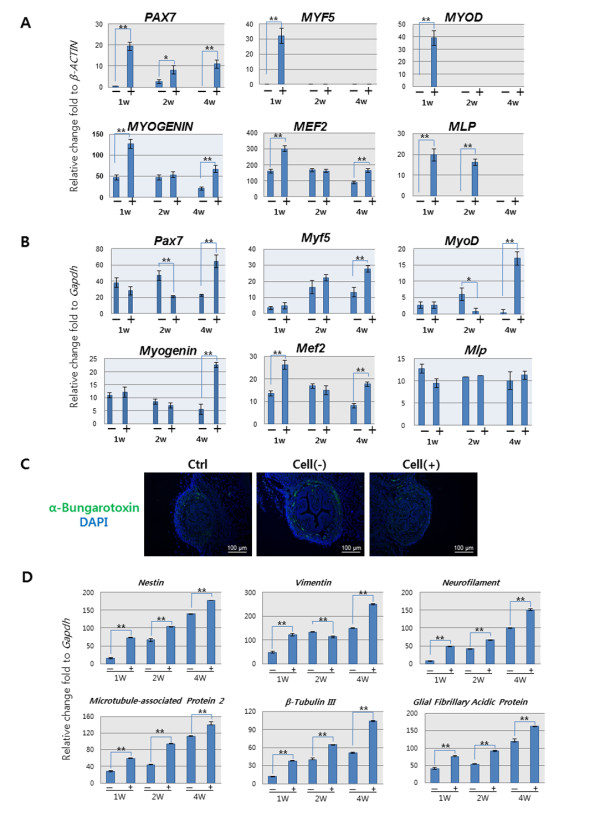
**Real-time PCR analysis of regenerated sphincter**. **(A) **Human gene expression was highest at the first week and gradually decreased with time (**P < 0.01; *P < 0.05). **(B) **Mouse gene expression was gradually increased with time (**P < 0.01; *P < 0.05). **(C) **Detection of the neuromuscular junction with α-Bungarotoxin staining. A regenerated neuromuscular junction was detected by the presence of an acetylcholine receptor. **(D) **Neurogenic marker gene expression was analyzed with real-time PCR. Significantly enhanced gene expression was seen in the cell-injected group compared with the non-injected group (**P < 0.01; *P < 0.05). --, pudendal neurectomy without cell injection; +, pudendal neurectomy with hAFSCs injection

After measurement of LPP and CP, the mice were sacrificed and the whole urethra was excised. The entire length of the urethra was about 7 mm and the rhabdosphincter was located about 6 mm distant from external urethral orifice (Figure 6A). H&E staining identified normal-appearing circular muscle mass regeneration over time at the urethral sphincter region in the Cell (+) group. In contrast, the Cell (-) group showed only scant muscle regeneration and an atrophic sphincter. These results were confirmed with IHC staining using MyoD antibody (Figure 6B). Real-time PCR analysis showed that the expression of genes related to early (*PAX7, MYF5 *and *MYOD*) and mid to late (*MYOGENIN, MEF2 *and *MLP*) myogenic differentiations corresponded correctly with time. Human gene expression was highest in the first week then gradually reduced (Figure 7A), whereas mouse gene expression gradually increased with time (Figure 7B). The IHC for neuromuscular junction formation showed that the Cell (+) group had a similar expression level of α-bungarotoxin for acetylcholine receptor as the normal control (Figure 7C). Real-time PCR showed neurogenic gene expression (Nestin, Vimentin, Neurofilament, Microtubule-associated Protein 2, β-Tubulin III, Glial Fibrillary Acidic Protein) was significantly higher (*P *< 0.05) in the Cell (+) group compared with the Cell (-) group (Figure 7D).

### Immunogenicity and tumorigenicity of hAFSCs

**Figure 8:**
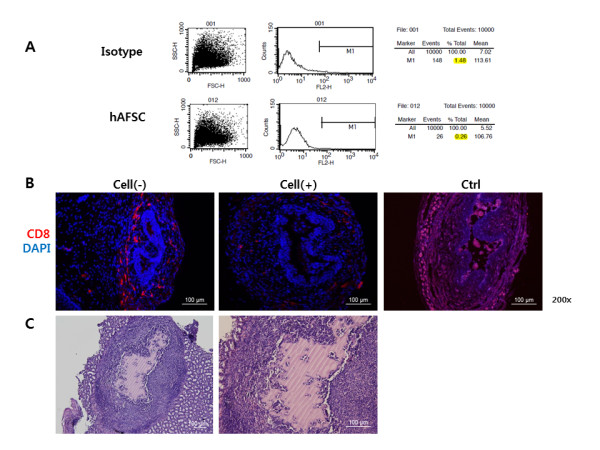
**Immunogenicity and tumorigenicity of hAFSCs**. **(A) **FACS analysis for HLA-DR expression. HLA-DR expression in hAFSCs was lower than isotype (negative control with PE-conjugated antibody). **(B) **IHC staining of a retrieved urethral sphincter one week after hAFSCs injection. The cell-injected animal showed scant CD8 lymphocyte aggregation, while the cell (-) and human fibroblast-injected animals had significant CD8 lymphocyte accumulation (200×). Cell (-), pudendal neurectomy without cell injection; Cell (+), pudendal neurectomy with cell treatment; Ctrl(+), positive control with human fibroblast injection (200×). **(C) **H&E stain of kidney tissue retrieved eight weeks after renal subcapsular injection of hAFSCs. No teratoma formation was found at the injection site (100× and 200×).

FACS analysis showed that HLA-DR expression in hAFSCs was lower (0.26% total) than that of the isotype control (1.48% total) (Figure 8A). IHC staining of the urethral sphincter tissue of hAFSC injected animals revealed scant CD8 lymphocyte aggregation at one week, whereas the Cell (-) and human fibroblast injected animals had significant CD8 lymphocyte accumulation (Figure 8B). Histologic analysis revealed no teratoma formation in tissues retrieved eight weeks after renal subcapsular injection of hAFSCs (Figure 8C).

## Discussion

Stem cell therapies have been proposed for SUI treatment as a way to overcome the limited efficacy and adverse reactions attributed to therapies involving bulking agents. However, most stem cell harvesting protocols require invasive procedures and/or result in harvesting of low cell numbers. Recently, hAFSCs have been proposed as a promising stem cell source for various cell therapies and tissue engineering. These cells can be obtained non-invasively and can differentiate into multiple cell lineages, such as adipocytes [7], osteoblasts [7], chondrocytes [13], renal cells [14], hepatocytes [4] and cardiomyocytes [15].

In the present study, we obtained a homogeneous genotypic cell profile using a double sorting procedure with C-KIT antibody. FACS analysis showed the hAFSCs were positive for mesenchymal stem cell markers, including SSEA4, CD44, CD73, CD90 and CD105, and the expression levels were similar to previous reports [7,16]. The hAFSCs showed negative expression for hematopoietic stem cell marker CD45. These results suggest that the hAFSCs were of the mesenchymal stem cell and not hematopoietic stem cell lineage.

When cultured in myogenic induction media, hAFSCs differentiated into muscle progenitor cells. During differentiation, expression of early myogenic differentiation markers (*PAX7 *and *MYOD*) decreased gradually, and expression of the middle (*DESMIN*) and late (*DYSTROPHIN*) differentiation markers increased over time.

Culture media containing 5-azaC and TGF-β induced a similar level of myogenic differentiation as did CM treatment. However, cell viability was significantly enhanced with CM treatment. These results suggest that hAFSCs have myogenic potential and that CM might be the best medium for induction of myogenic differentiation.

We also evaluated the therapeutic feasibility of periurethral injection of hAFSCs in an SUI animal model. When hAFSCs were injected into the animal, IHC staining with HuNu confirmed that the injected cells were able to survive in the host environment. They integrated into the mouse sphincter muscle layer and survived within these *in vivo *conditions for 14 days. Real-time PCR gave us valuable information on the interaction between human cells and mouse cells. Human myogenic gene expression gradually decreased over time, while mouse gene expression steadily increased. These results indicate the grafted hAFSCs might have undergone *in situ *myogenic differentiation and induced host muscle regeneration. These findings are similar to other reports of human stem cell transplantation into animals [17-19]. The details underlying the specific mechanism of action need to be investigated.

Clinically, SUI can occur acutely or chronically in humans. Chronic SUI, which usually occurs in the female population, is commonly caused by weakness of urethral sphincter muscle owing to vaginal delivery, for example. Meanwhile, acute SUI can be induced by urethral sphincter muscle resection or atrophy, after prostate surgery in males or perineal trauma. In all cases, we can expect improvements of incontinence if urethral sphincter muscle is restored morphologically and functionally. We observed sphincter muscle atrophy after bilateral pudendal nerve transection in this study and we confirmed the regeneration of urethral sphincter muscle after cell injection *in vivo*. Although our animal model is closer to acute SUI rather than chronic SUI, we believe this therapeutic method can improve both chronic and acute SUI by functional restoration of urethral sphincter muscle.

The wide use of human stem cells for therapeutic application has prompted the search for non-invasive methods for tracking injected cells. For example, Delo *et al. *developed an MRI-based cell tracking method [20], and were able to detect injected hAFSCs up to four weeks. However, this method requires expensive high resolution MRI and has a potential radiation hazard. In the present study, we established an optical imaging-based cell tracking method by labelling cells with MNPs@SiO2 (RITC). AFSCs were labeled with nanoparticles without signs of cytotoxicity. The labeled cells were detected for up to 10 days after injection using optical imaging. At Day 14, the signal strength was under the detection range. These results suggest that MNPs@SiO2 (RITC) can be used for noninvasive *in vivo *tracking of injected hAFSCs. A limitation of optical imaging is that the detector could not catch the signal when the signal strength was under the detection range. We assumed the signal strength at Day 14 was out of the optical imaging range. We, therefore, performed real-time PCR to confirm longer detection of muscle regeneration.

Histological and IHC analysis showed that periurethral injection of hAFSCs into denervated urethral sphincter stimulated normal-appearing sphincter muscle regeneration over time. These results correlated well with the real-time PCR analysis for myogenic gene expressions over time. The functional analysis of the sphincters showed that LPP and CP of the hAFSC injected group were restored to nearly normal values, while the Cell (-) group values remained low throughout the study period. These results indicate that periurethral injection of hAFSCs into a denervated urethral sphincter can restore apparently normal urethral sphincter histology and function.

Restoration of sphincter function requires regeneration of a neuronal component (neuromuscular junction and nerve regeneration) as well as muscle regeneration. In this study, we found similar expression levels in the Cell (+) group and the normal control, whereas the Cell (-) group showed a higher expression level. This indicates that the transplanted hAFSCs can control damaged tissue regeneration by harmonizing the physical environment [21]. Over-proliferation or unwanted differentiation can cause malignant formation. In addition, significantly enhanced neurogenic gene expression was seen in the Cell (+) group compared with the Cell (-) group. This result suggests that injected cells can have a physiologic effect on the neuromuscular junction re-formation and nerve regeneration.

Recently, several reports have suggested that stem cells may have a low immunogenicity and immunomodulatory function [22,23]. In this study, we found that hAFSCs have a lower expression of HLA-DR compared with isotope control. Furthermore, injection of hAFSCs into ICR mice also did not stimulate CD8+ T cell infiltration into the injected area. These findings suggest that hAFSCs have an immune tolerance and/or immunosuppression effect, similar to that reported for other stem cells. When injected into the renal subcapsule, hAFSCs did not cause teratomas after eight weeks. This result supports the use of hAFSCs as a safe for cell therapeutic application in terms of tumorigenicity.

The limitations of this study include the lack of identification of a precise mechanism for a paracrine effect, nerve regeneration, and the inability to track the metabolism or fate of the injected cells *in vivo*.

## Conclusions

We were able to isolate homogenous hAFSCs possessing mesenchymal stem cell characteristics and these cells had the potential to differentiate into a myogenic lineage *in vitro*. We established a successful non-invasive *in vivo *cell tracking procedure and used it to follow hAFSCs injected into the denervated urethral sphincter. These cells promoted regeneration of urethral sphincter muscle, morphologically and functionally similar to the normal urethral sphincter, apparently through *in situ *differentiation and host cell stimulation by the hAFSCs. The xenograft hAFSC transplantation did not cause any immune response or tumor formation at the injection site. Therefore, hAFSCs appear to be a promising cell source for SUI stem cell therapy.

## Abbreviations

5-azaC: 5-aza-20-deoxycytidine; CM: conditioned medium; CP: closing pressure; DAPI: 4,6-diamidino-2-phenylindole 2HCl staining; DMEM: (Dulbecco's) Modified Eagle's Medium; FACS: fluoresecence-activated cell sorting; hAFSCs: human amniotic fluid stem cells; H&E: hematoxylin and eosin; HuNu: human nuclear specific antibody; ICC: immunocytochemical; ICR: imprinting control region; IHC: immunohistochemical; LPP: leak point pressure; MACS: magnetic activated cell sorting system; MSC: mesenchymal stem cell; PBS: phosphate-buffered saline; PCR: polymerase chain reaction; PE: phycoerythrin; RITC: rhodamine B isothiocyanate; SD: standard deviation; SUI: stress urinary incontinence; TGF-β: transforming growth factor-β.

## Competing interests

The authors declare that they have no competing interests.

## Authors' contributions

BSK and SYC drafted the manuscript. JKL, HJL and BSK participated in data collection and analysis. JSB, HYC and SS participated in data interpretation. AA and JJY participated in the conception and design of the study. TGK designed the general study. All authors reviewed and approved the final manuscript.

## Pre-publication history

The pre-publication history for this paper can be accessed here:

http://www.biomedcentral.com/1741-7015/10/94/prepub
